# Supplementary material for: Optimal timing of nirmatrelvir/ritonavir treatment after COVID-19 symptom onset or diagnosis: target trial emulation
Source: Nat Commun. 2023 Dec 16;14:8377. doi: 10.1038/s41467-023-43706-0 (PMC10725470; doi:10.1038/s41467-023-43706-0)

Supplementary Materials

Supplementary Table 1. Summary of the study protocol of emulating a target trial using observational data on the study outcome of all-cause mortality or hospitalization

Supplementary Table 2. Summary of the study protocol of emulating a target trial using observational data on the study outcome of viral burden rebound

Supplementary Figure 1: Cumulative incidence plot for the study outcome of 28-day all-cause mortality or hospitalization among early (0-1 day) and late (≥2 days) initiators

Supplementary Figure 2: Ct value trajectory plot of nirmatrelvir/ritonavir users over the first 28 days of follow-up among early (0-1 day) and late (≥2 days) initiators

Supplementary Figure 3: Cumulative incidence plot for the study outcome of VBR among early (0-1 day) and late (≥2 days) initiators

Supplementary Table 1. Summary of the study protocol of emulating a target trial using observational data on the study outcome of all-cause mortality or hospitalization

Protocol component	Target trial specification	Emulation using observational data
Eligibility criteria	Adults aged ≥18 years, with confirmed SARS-CoV-2 infection diagnosis and nirmatrelvir/ritonavir use during the study inclusion period<br><br>Patients are excluded if:<br>• Hospitalized or dead on or before the index date<br>• With severe renal impairment (estimated glomerular filtration rate [eGFR] <30 mL/min/1.73m^2, dialysis, or renal transplantation)<br>• With severe liver impairment (cirrhosis, hepatocellular carcinoma, or liver transplantation)<br>• With drug contraindications to nirmatrelvir/ritonavir<br>• Initiated molnupiravir treatment on or before the index date<br><br>Index date is defined as that of SARS-CoV-2 infection diagnosis or symptom onset, whichever occurred earlier.	Same as specification, and patients are also excluded if:<br>• No recorded date of SARS-CoV-2 infection diagnosis or symptom onset<br>• The recorded date of nirmatrelvir/ritonavir prescription is before that of SARS-CoV-2 infection diagnosis or symptom onset<br>• With record of molnupiravir prescription (i.e. ever used before the index date or during the 28-day follow-up period)<br><br>Date of confirmed SARS-CoV-2 infection diagnosis is the date of first positive reverse transcription polymerase chain reaction (RT-PCR) or rapid antigen test (RAT) result during the study inclusion period.
Treatment strategy	Early initiation: prescription of nirmatrelvir/ritonavir within 1 day from the index date (Day 0-1, i.e. on the same day of SARS-CoV-2 infection diagnosis or symptom onset, or the next calendar date)	Same as specification

	Late initiation: prescription of nirmatrelvir/ritonavir on Day 2 or after from the index date	
Assignment procedures	Patients are randomly assigned to receive early versus late initiation nirmatrelvir/ritonavir.	Patients are classified into early or late initiation groups based on their timing of recorded nirmatrelvir/ritonavir prescription from the index date.<br><br>Random assignment of the timing of nirmatrelvir/ritonavir initiation is assumed by the adoption of the inverse probability weighting-cloning-censoring-inverse probability of censoring weighting (IPW-Cloning-Censoring-IPCW) analysis.
Outcomes	28-day all-cause mortality or all-cause hospitalization	Same as specification
Follow-up period	Patients are observed from the index date (time zero) until that of hospital admission, registered death, 28 days after the index date, or the administrative end of the follow-up period (12th February 2023), whichever the earliest.	Same as specification
Causal contrasts of interest	Intention-to-treat effect<br>Per-protocol effect	Observational analogue of the per-protocol effect
Analysis plan	Intention-to-treat analysis<br>Per-protocol analysis: patients are censored when they deviate from their respective treatment strategies (early or late initiation)	Same as per-protocol analysis, adjusted for baseline confounders

Supplementary Table 2. Summary of the study protocol of emulating a target trial using observational data on the study outcome of viral burden rebound

<table>
<tr><th>Protocol component</th><th>Target trial specification</th><th>Emulation using observational data</th></tr>
<tr><td>Eligibility criteria</td><td>Adults aged ≥18 years, with confirmed SARS-CoV-2 infection diagnosis and nirmatrelvir/ritonavir use during the study inclusion period

Patients are excluded if:
Dead on or before the index date
With severe renal impairment (estimated glomerular filtration rate [eGFR] <30 mL/min/1.73m^2, dialysis, or renal transplantation)
With severe liver impairment (cirrhosis, hepatocellular carcinoma, or liver transplantation)
With drug contraindications to nirmatrelvir/ritonavir
Initiated molnupiravir treatment on or before the index date
Index date is defined as that of SARS-CoV-2 infection diagnosis or symptom onset, whichever occurred earlier.</td><td>Same as specification, and patients are also excluded if:
No recorded date of SARS-CoV-2 infection diagnosis or symptom onset
The recorded date of nirmatrelvir/ritonavir prescription is before that of SARS-CoV-2 infection diagnosis or symptom onset
With record of molnupiravir prescription (i.e. ever used before the index date or during the 28-day follow-up period)
Without at least one Ct value measurement within 14 days prior to the index date
Date of confirmed SARS-CoV-2 infection diagnosis is the date of first positive reverse transcription polymerase chain reaction (RT-PCR) or rapid antigen test (RAT) result during the study inclusion period.</td></tr>
<tr><td>Treatment strategy</td><td>Early initiation: prescription of nirmatrelvir/ritonavir within 1 day from the index date (Day 0-1, i.e. on the same day of SARS-CoV-2 infection diagnosis or symptom onset, or the next calendar date)</td><td>Same as specification</td></tr>
</table>

	Late initiation: prescription of nirmatrelvir/ritonavir on Day 2 or after from the index date	
Assignment procedures	Patients are randomly assigned to receive early versus late initiation nirmatrelvir/ritonavir.	Patients will be classified into early or late initiation groups based on their timing of recorded nirmatrelvir/ritonavir prescription from the index date.<br><br>Random assignment of the timing of nirmatrelvir/ritonavir initiation is assumed following 1) inverse probability weighting on pre-specified baseline characteristics of patients, namely age, sex, living area, Charlson Comorbidity Index, symptomatic presentation, concomitant use of corticosteroids, immunocompromised state, healthcare utilization in the past year, previous SARS-CoV-2 infection, COVID-19 vaccination status, date of SARS-CoV-2 infection diagnosis, type of care received, and type of viral test for case detection, 2) cloning, 3) censoring and 4) inverse probability censoring-weighting.
Outcomes	Viral burden rebound (VBR) within 28 days from the index date<br><br>VBR is defined as a reduction in Ct value (provided by SARS-CoV-2 quantitative RT-PCR assays) between two consecutive measurements ≥3, and such decrease was sustained in at least the immediately subsequent Ct measurement (ΔCt = Ct_[before] – Ct_[after 1] ≥3 and ΔCt = Ct_[before] – Ct_[after 2] ≥3).	Same as specification
Follow-up period	Patients are observed from the index date (time zero) until that of	Same as specification

	VBR, 28 days after the index date, or the administrative end of the follow-up period (12th February 2023), whichever the earliest.<br><br>Patients are censored on the date of registered death (competing event).	
Causal contrasts of interest	Intention-to-treat effect<br>Per-protocol effect	Observational analogue of the per-protocol effect
Analysis plan	Intention-to-treat analysis<br>Per-protocol analysis: patients are censored when they deviate from their respective treatment strategies (early or late initiation)	Same as per-protocol analysis, adjusted for baseline confounders

Supplementary Figure 1: Cumulative incidence plot for the study outcome of 28-day all-cause mortality or hospitalization among early (0-1 day) and late (≥2 days) initiators

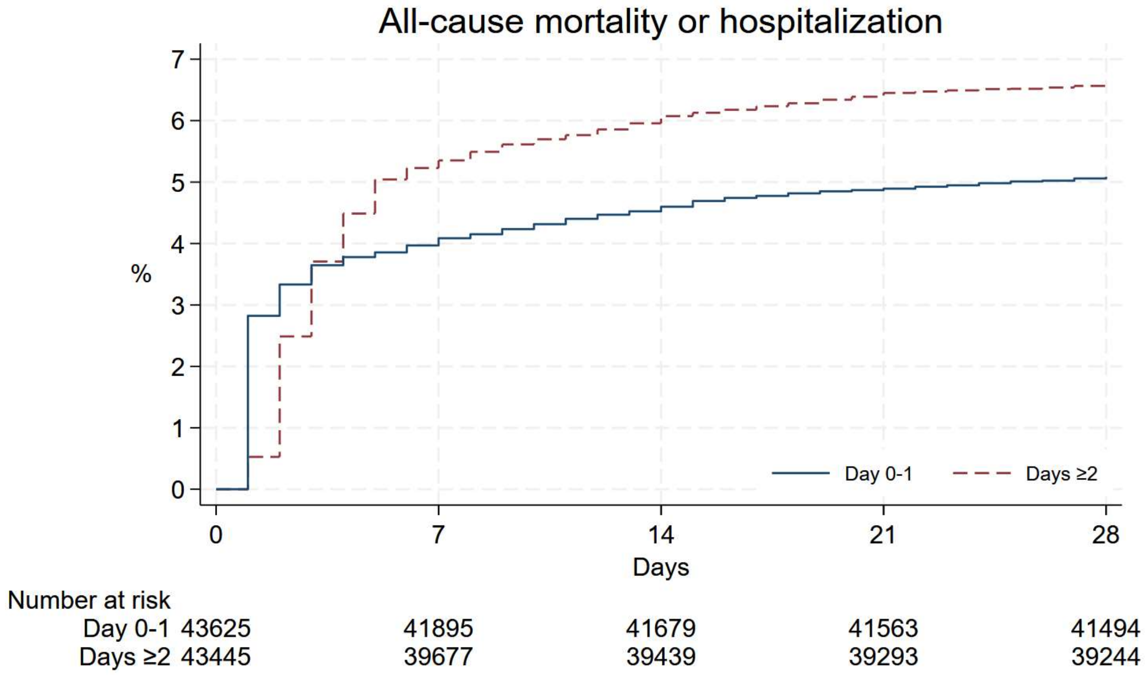

Supplementary Figure 2: Ct value trajectory plot of nirmatrelvir/ritonavir users over the first 28 days of follow-up among early (0-1 day) and late (≥2 days) initiators

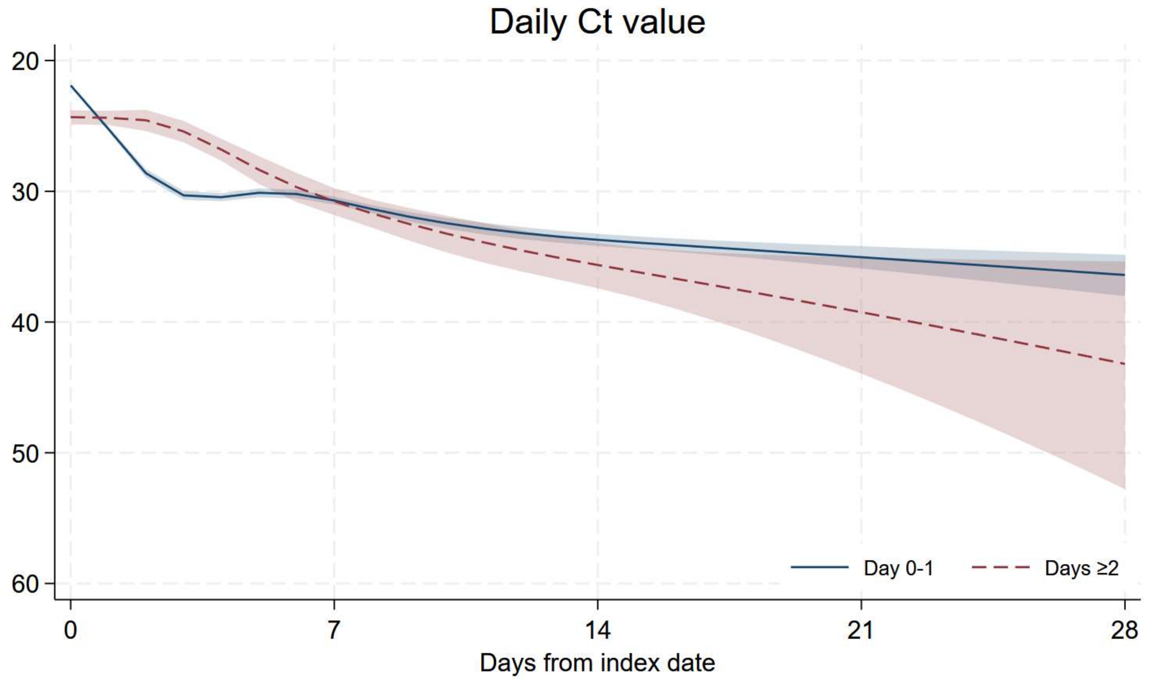

Supplementary Figure 3: Cumulative incidence plot for the study outcome of VBR among early (0-1 day) and late (≥2 days) initiators

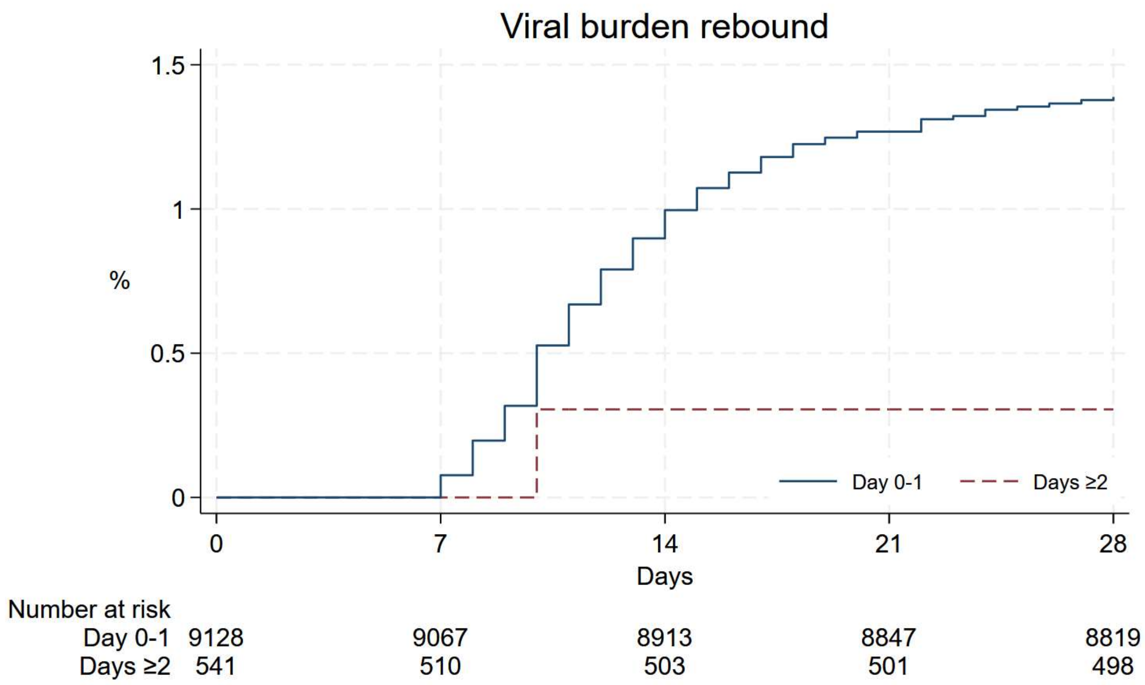

Supplement: Supplementary file 1 — Supplementary material [file 41467_2023_43706_MOESM1_ESM.pdf]
