# Supplementary material for: Optimal timing of nirmatrelvir/ritonavir treatment after COVID-19 symptom onset or diagnosis: target trial emulation
Source: Nat Commun. 2023 Dec 16;14:8377. doi: 10.1038/s41467-023-43706-0 (PMC10725470; doi:10.1038/s41467-023-43706-0)
Supplement: Supplementary file 3 — Reporting Summary [file 41467_2023_43706_MOESM3_ESM.pdf]

nature portfolio

Corresponding author(s): Joseph Wu

Last updated by author(s): Nov 6, 2023

Reporting Summary

Nature Portfolio wishes to improve the reproducibility of the work that we publish. This form provides structure for consistency and transparency in reporting. For further information on Nature Portfolio policies, see our Editorial Policies and the Editorial Policy Checklist.

Statistics

For all statistical analyses, confirm that the following items are present in the figure legend, table legend, main text, or Methods section.

n/a	Confirmed	
☐	☒	The exact sample size (*n*) for each experimental group/condition, given as a discrete number and unit of measurement
☒	☐	A statement on whether measurements were taken from distinct samples or whether the same sample was measured repeatedly
☐	☒	The statistical test(s) used AND whether they are one- or two-sided<br>*Only common tests should be described solely by name; describe more complex techniques in the Methods section.*
☐	☒	A description of all covariates tested
☐	☒	A description of any assumptions or corrections, such as tests of normality and adjustment for multiple comparisons
☐	☒	A full description of the statistical parameters including central tendency (e.g. means) or other basic estimates (e.g. regression coefficient) AND variation (e.g. standard deviation) or associated estimates of uncertainty (e.g. confidence intervals)
☐	☒	For null hypothesis testing, the test statistic (e.g. *F*, *t*, *r*) with confidence intervals, effect sizes, degrees of freedom and *P* value noted<br>*Give P values as exact values whenever suitable.*
☒	☐	For Bayesian analysis, information on the choice of priors and Markov chain Monte Carlo settings
☒	☐	For hierarchical and complex designs, identification of the appropriate level for tests and full reporting of outcomes
☒	☐	Estimates of effect sizes (e.g. Cohen's *d*, Pearson's *r*), indicating how they were calculated

Our web collection on statistics for biologists contains articles on many of the points above.

Software and code

Policy information about availability of computer code

Data collection	All data transformation and analyses were performed in STATA (version 18). Analytic codes can be found at: https://github.com/IvanAuHKU/COVID-nirmatrelvir-ritonavir-timing
Data analysis	All data transformation and analyses were performed in STATA (version 18). Analytic codes can be found at: https://github.com/IvanAuHKU/COVID-nirmatrelvir-ritonavir-timing

For manuscripts utilizing custom algorithms or software that are central to the research but not yet described in published literature, software must be made available to editors and reviewers. We strongly encourage code deposition in a community repository (e.g. GitHub). See the Nature Portfolio guidelines for submitting code & software for further information.

Data

Policy information about availability of data

All manuscripts must include a data availability statement. This statement should provide the following information, where applicable:

- Accession codes, unique identifiers, or web links for publicly available datasets
- A description of any restrictions on data availability
- For clinical datasets or third party data, please ensure that the statement adheres to our policy

The clinical outcome data and vaccination records were extracted from the Hospital Authority database in Hong Kong and data on confirmed cases of SARS-CoV-2 infection were extracted from the eSARS data provided by the Centre for Health Protection (Department of Health, The Government of the Hong Kong Special Administrative Region). The data custodians (the Hospital Authority and the Department of Health) provided the underlying individual patient data to The University

of Hong Kong for the purpose of performing scientific research for the study. Restrictions apply to the availability of these data, which were used under licence of the Hospital Authority and the Department of Health for this study. The authors cannot transmit or release the data, in whole or in part in whatever form or media, or to any other parties or place outside Hong Kong; and the authors fully comply with the duties under the laws of Hong Kong relating to the protection of personal data including those under the Personal Data (Privacy) Ordinance and its principles in all aspects.

Research involving human participants, their data, or biological material

Policy information about studies with human participants or human data. See also policy information about sex, gender (identity/presentation), and sexual orientation and race, ethnicity and racism.

Reporting on sex and gender	Sex was considered in the study design, and sex of participants was determined based on self-report. Sex distribution and sex-stratified results was reported.
Reporting on race, ethnicity, or other socially relevant groupings	No information on race, ethnicity, or other socially relevant groupings were reported in this study. These information were not included in the database.
Population characteristics	Baseline characteristics (age, sex, regions, time of SARS-CoV-2 infection, pre-existing comorbidities, and COVID-19 vaccination status) of nirmatrelvir/ritonavir users who initiated early (day 0-1) and late (days ≥2) in mortality or hospitalization analysis and viral burden rebound analysis are reported in Table 1.
Recruitment	Population-based electronic health records database
Ethics oversight	This study was approved by the institutional review board of the University of Hong Kong / Hospital Authority Hong Kong West Cluster (reference no. UW 20-493). Individual patient-informed consent was not required for this retrospective cohort study using anonymized data.

Note that full information on the approval of the study protocol must also be provided in the manuscript.

Field-specific reporting

Please select the one below that is the best fit for your research. If you are not sure, read the appropriate sections before making your selection.

[x] Life sciences   [ ] Behavioural & social sciences   [ ] Ecological, evolutionary & environmental sciences

For a reference copy of the document with all sections, see nature.com/documents/nr-reporting-summary-flat.pdf

Life sciences study design

All studies must disclose on these points even when the disclosure is negative.

Sample size	A total of 87,070 nirmatrelvir/ritonavir users who had confirmed diagnoses of SARS-CoV-2 infection between 16th March 2022 and 15th January 2023 were included in this analysis.
Data exclusions	We excluded patients with missing index dates, who were aged <18 years at index date, whose recorded date of nirmatrelvir/ritonavir prescription was before their index date, who were dead on or before the date of nirmatrelvir/ritonavir initiation, who initiated nirmatrelvir/ritonavir beyond 5 days from the index date, or who had ever been prescribed molnupiravir before the index date or during the follow-up period (as oral antiviral use may impact VBR). Patients with contraindications to nirmatrelvir/ritonavir were also excluded, namely those with severe renal (eGFR <30 mL/min/1.73m2, dialysis, or renal transplantation) or liver disease (cirrhosis, hepatocellular carcinoma, or liver transplantation) at baseline, or those with drug contraindications to the concomitant use of nirmatrelvir/ritonavir.<br><br>For the target trial emulation specific to all-cause mortality or hospitalization, we also excluded patients who were hospitalized on or before the recorded date of nirmatrelvir/ritonavir prescription, and those who were dead or hospitalized within two days from the index date.<br><br>For the target trial emulation on the outcome of viral burden rebound, only patients with at least one Ct value measurement before or during nirmatrelvir/ritonavir treatment and at least one Ct value measurement after the end of treatment course were included for analysis.
Replication	Interested parties could contact the the Hospital Authority of the Hong Kong Special Administrative Region and the Centre for Health Protection (Department of Health, The Government of the HKSAR) for access to anonymised clinical outcome data and vaccination records, respectively.<br><br>Please note that the above data, which forms the core of our analysis, is necessary for our full results to be replicated.
Randomization	Randomization was not applicable to this retrospective cohort study.
Blinding	Blinding was not applicable to this retrospective cohort study.

Reporting for specific materials, systems and methods

We require information from authors about some types of materials, experimental systems and methods used in many studies. Here, indicate whether each material, system or method listed is relevant to your study. If you are not sure if a list item applies to your research, read the appropriate section before selecting a response.

Materials & experimental systems

n/a	Involved in the study
☒	☐ Antibodies
☒	☐ Eukaryotic cell lines
☒	☐ Palaeontology and archaeology
☒	☐ Animals and other organisms
☒	☐ Clinical data
☒	☐ Dual use research of concern
☒	☐ Plants

Methods

n/a	Involved in the study
☒	☐ ChIP-seq
☒	☐ Flow cytometry
☒	☐ MRI-based neuroimaging
